# Non-hepatitis-associated mixed cryoglobulinemia with polyclonal plasma cells disease and alcoholic cirrhosis: A rare case report

**DOI:** 10.3389/fmed.2023.1014261

**Published:** 2023-01-25

**Authors:** Jiao Luo, Cheng Liu, Qing-Jian Lv, Ting He, Xing Qiang, Yi Li, Qi-Mi Huang, Jia-Lin He

**Affiliations:** ^1^Department of Gastroenterology, Xinqiao Hospital, Army Medical University, Chongqing, China; ^2^Department of Nephrology, Xinqiao Hospital, Army Medical University, Chongqing, China; ^3^Department of Hematology, Xinqiao Hospital, Army Medical University, Chongqing, China; ^4^Department of Clinical Laboratory, Xinqiao Hospital, Army Medical University, Chongqing, China

**Keywords:** mixed cryoglobulinemia, cryoglobulins, alcoholic liver cirrhosis, renal impairment, polyclonal plasma cells disease

## Abstract

Mixed cryoglobulinemia refers to the serum presence of a variety of cryoglobulins, which are defined as immunoglobulins that precipitate at temperatures of < 37°C. The most common cause of mixed cryoglobulinemia is hepatitis C virus (HCV), while other infections, including hepatitis B virus (HBV) and HIV infections, and lymphoproliferative and autoimmune disorders have also been associated with the disease. We reported a rare case of type II–III mixed cryoglobulinemia caused by alcoholic cirrhosis. We need to increase the awareness of and facilitate the early identification of mixed cryoglobulinemia in our clinical study when encountering a patient with liver cirrhosis combined with renal impairment so that treatment can begin early to improve the success rate of therapy and reduce the fatality rate in a potentially life-saving therapy.

## Introduction

Cryoglobulinemia is defined as the presence of cryoglobulins, which are immunoglobulins that can precipitate at temperatures of < 37°C in the serum. The disease is associated with many illnesses, such as infections, autoimmune disorders, and malignancies. The most common cause of cryoglobulinemia is hepatitis C virus (1). The composition of cryoglobulins is heterogeneous. The most commonly used classification is Brouet's classification (2) from 1974 based on the clonality and type of immunoglobulins. Type I comprises monoclonal immunoglobulin (generally either IgM or IgG). Types II and III are classified as mixed cryoglobulinemia because they consist of both IgG and IgM components. Type II cryoglobulins are a mixture of monoclonal IgM and polyclonal IgG and Type III cryoglobulins are a mixture of polyclonal IgM and IgG. This classification system also has limitations and does not take into account the atypical features of cryoglobulinemia. Another subset of cryoglobulinemia, known as type II-III mixed cryoglobulinemia, might be an intermediate evolution from type III to type II mixed cryoglobulinemia (3). Several studies proposed that cryoglobulins with or without oligoclonal IgM components, caused by trace polyclonal immunoglobulin reactions, are commonly referred to as type II–III (4, 5) or biclonal cryoglobulins (6, 7). Most cases of mixed cryoglobulinemia have a known underlying cause. The most common cause of mixed cryoglobulinemia is hepatitis C virus (HCV), while other infections, including hepatitis B virus (HBV) and HIV infections, and lymphoproliferative and autoimmune disorders have also been associated with the disease. A previous study reported that mixed cryoglobulins made of heterogeneous immunoglobulins without monotypic components were mostly associated with established cirrhosis (8), and others found that alcoholic cirrhosis is a rare cause of cryoglobulinemia (3). Treatment should be modulated according to the underlying etiopathogenesis and the severity of clinical presentation. There are three broad strategies in the treatment of cryoglobulinemia: conventional immunosuppression, antiviral treatments, and biological therapies, and studies found that both combined or sequential antiviral therapies and targeted biological treatments might be more effective than monotherapy (9, 10).

## Case description

A 35-year-old male patient was admitted with fever and bloating for 2 weeks. Three years ago, the patient was diagnosed with alcoholic cirrhosis after consumption of a lot of liquor (about 500–750 ml daily) for more than 10 years and had undergone partial splenic embolization (PSE; ~40%) 3 years back for low platelets. He was on hepatocyte growth-promoting factors enteric-coated capsules regularly.

He had fever and bloating accompanied by dizziness, malaise, cough, and runny nose. Except for the increased abdominal circumference and bloating, the other symptoms of him have disappeared after the corresponding treatments were received. Investigations revealed hemoglobin of 78 g/L (normal 130–175 g/L), serum brain natriuretic peptide (BNP) of 1,130 pg/ml (normal 5.0–100 pg/ml), and serum creatinine of 120.7 μmol/L (normal 45–105 μmol/L). Serology was negative for HBV DNA, HCV RNA, antinuclear antibody (ANA), anti-mitochondrial antibodies (AMA), anti-smooth muscle antibodies (ASMA), anti-liver kidney microsome type I (anti-LKM1), Ro/La autoantibodies, anti-ds DNA, alpha-fetal protein (AFP), carcinoembryonic antigen (CEA), and rheumatoid factor (RF). The computed tomography showed characteristic manifestations of cirrhosis, splenomegaly, and ascites.

We concluded that the patient was in the decompensated stage of alcoholic cirrhosis with esophagogastric varices and ascites and of renal insufficiency. Therefore, transjugular intrahepatic portosystemic shunt (TIPS) surgery was performed to prevent variceal hemorrhage and other related complications of portal hypertension, and the pressure in the portal vein was measured. The portal vein pressure decreased from 53 cm H_2_O preoperatively to 27 cm H_2_O postoperatively (normal 13–24 cm H_2_O).

**Figure 1:**
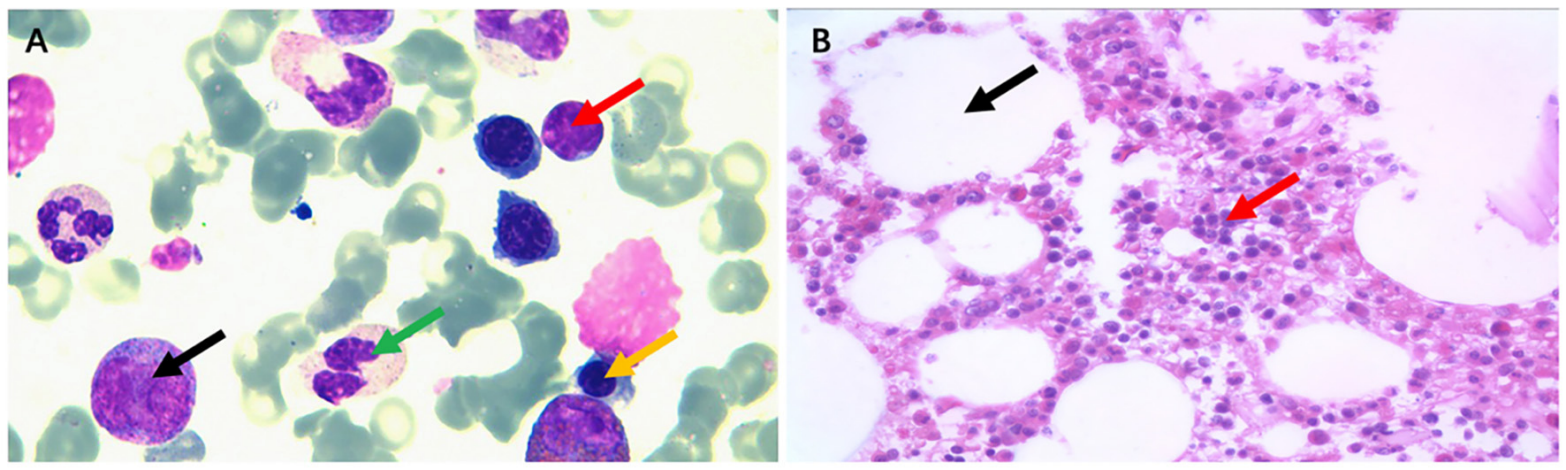
The bone marrow cytology and pathological biopsy. The bone marrow cytology **(A)** revealed active proliferation of hematopoietic cells including granulocyte, erythroid, and megakaryocyte lineages (black arrows point to promyelocytes, green arrows point to mature granulocytes, yellow arrows point to the immature red blood cells in the middle and late stages, and red arrows point to mature lymphocytes). **(B)** The biopsy of the bone marrow showed that myeloproliferation is normal and hematopoietic tissue is 50% (black arrows indicate adipose tissue and red arrows indicate hematopoietic tissue).

He had a paroxysmal dry cough, fever, shortness of breath, facial edema, and bilateral lower edema extremity on the 5th postoperative day. The patient presented with the classic Meltzer triad (asthenia, arthralgia, and skin purpura) but with no symptoms of sensorimotor polyneuropathy, and gradually developed the failure of the heart, kidney, and liver. Investigations revealed BNP of 2,210 pg/ml (normal 5.0–100 pg/ml), estimated glomerular filtration rate (EGFR) of 55 ml/min (normal >90 ml/min), serum creatinine of 141.2 μmol/L (normal 5.0–100 pg/ml), 24-h urinary protein quantification of 6.32 g/24 h urine (normal 0–1.2 g/24 h urine), 24-h urine microalbumin quantification of 3,838 mg/24 h urine (normal 0–30 mg/24 h urine), hemoglobin of 59 g/L (normal 130–175 g/L), platelets of 53 × 10^9^/L (normal 100–300 × 10^9^/L), thrombin time (TT) of 20.2 s (normal 10.3–16.6 s), plasma prothrombin time (PT) of 15.1 s (normal 9.4–12.5 s), activated partial thrombopladtin time (APTT) of 46.3 s (normal 25.1–36.5 s), albumin of 19 g/L (normal 40–55 g/L), serum complement C4 of 5.37 mg/dL (normal 16–38 mg/dL), serum complement C3 of 0.35 g/L (normal 0.9–2.1 g/L), immunoglobulin A (Ig A) of 10.7 g/L (normal 0.7~−4.0 g/L), immunoglobulin G (Ig G) of 20.5 g/L (normal 7–15 g/L), and immunoglobulin M (Ig M) of 1.28 g/L (normal 0.4–2.6 g/L). The laboratory results are that HBV DNA is < 20 IU/ml (normal < 20 IU/ml) and HCV RNA is < 50 U/ml (normal < 50 IU/ml). The bone marrow cytology revealed active proliferation of hematopoietic cells, including granulocyte, erythroid, and megakaryocyte lineages (Figure 1A), and the pathological biopsy of the bone marrow showed that myeloproliferation is normal (Figure 1B). Bone marrow examination revealed 18.06% infiltration by a mix of partial cKappa, cLambda, CD45im/CD38bri, CD138, and CD19 positive polyclonal B lymphocytes and polyclonal plasma cells.

**Figure 2:**
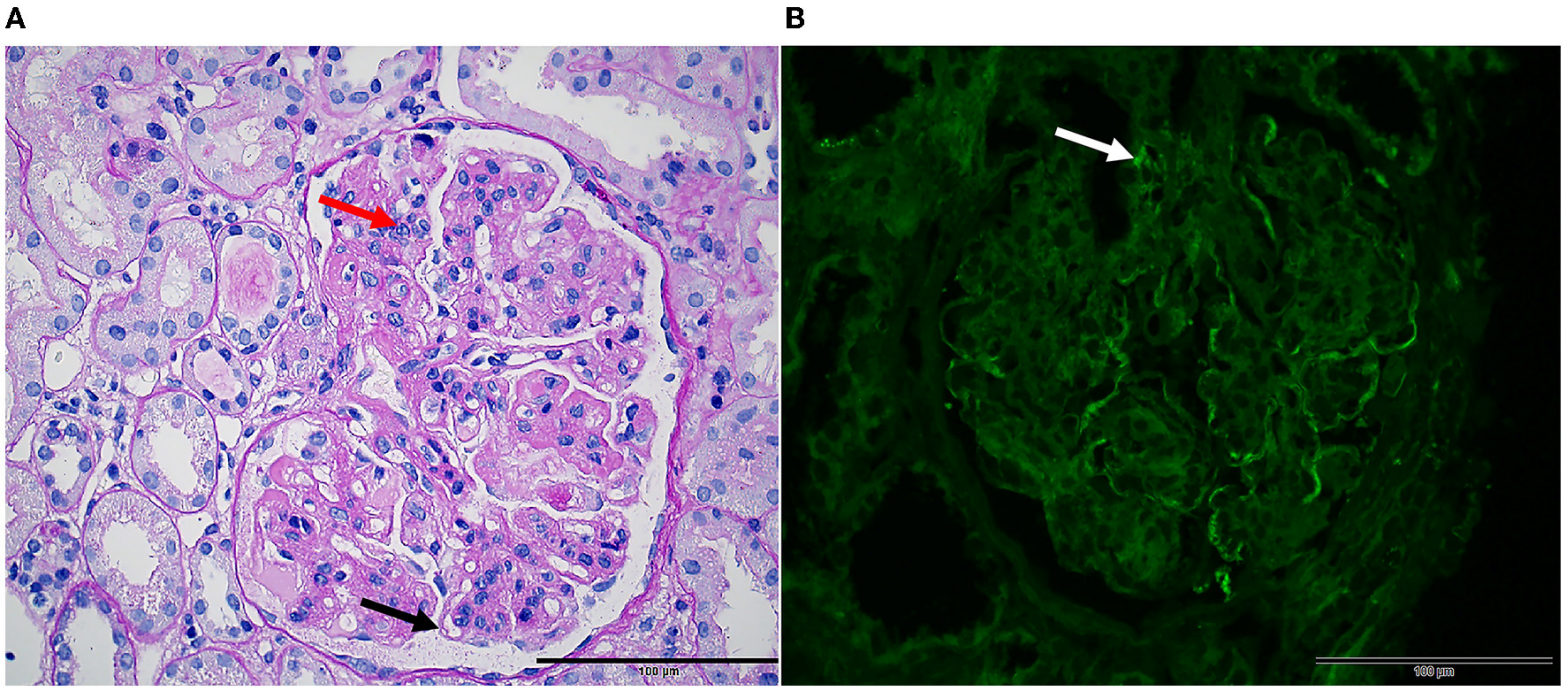
Renal pathology in mixed cryoglobulinemia. Light microscopy ( × 10). **(A)** PAS stain shows membranoproliferative glomerulonephritis characterized by nodular lobulated changes in glomeruli, segmentally thickened and double-contoured appearance of the glomerular basement membrane (black arrow), diffuse intracapillary cell accumulation, and inflammatory cell infiltration (red arrow). **(B)** Immunofluorescence studies showing Immunoglobulin G deposit in vascular loops with line-like (white arrow).

**Figure 3:**
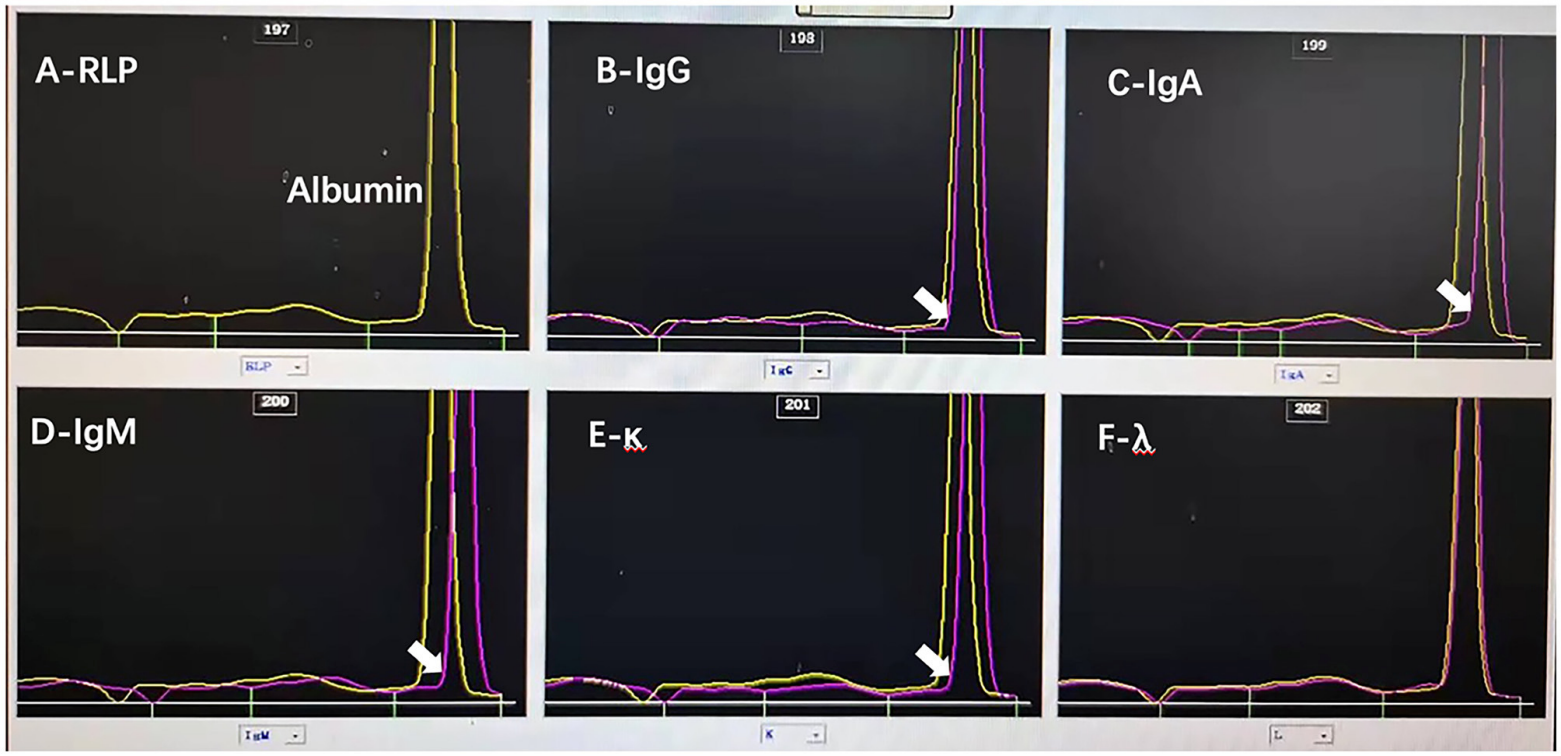
The results of capillary electrophoresis directing analysis of serum by capillary electrophoresis with detection at 200 nm. Polyclonal IgG κ and IgA κ proteins are identified in the serum of the patient by using fully automated capillary electrophoresis. The ELP pattern of **(A)** (yellow line) is the original serum line which is the electrophoresis result of the original sample. **(B)** The serum contains an IgG polyclonal protein. **(C)** The serum contains an IgA polyclonal protein. **(D)** The serum does not contain an IgM protein. **(E)** The serum contains a kappa chain, and **(F)** the serum does not contain a light chain.

A renal biopsy was performed, and it showed membranoproliferative glomerulonephritis and was considered pathological changes of cryoglobulinemic glomerulonephritis (Figure 2). Immunofluorescence staining was positive for C1q, IgA1, IgG1, IgM, fibrinogen, predominantly kappa and lambda light chain, C3, and C4c. Immunohistochemically, the glomerular capillary loop exhibited a highly diffuse expression of CD-68 protein, showed negative for the presence of idiopathic membranous nephropathy marker (phospholipase A2 receptor, PLA2R), and indicated the absence of primary glomerular disease markers CD3/CD20/CD68/CD38. To further confirm the diagnosis of cryoglobulinemia, mixed cryoglobulins were identified in peripheral blood. The protein quantification of mixed cryoglobulin was measured as 112 mg/L using the biuret method. It was found to be mainly composed of IgG κ and IgA κ using the instrument in our experiment, a model CAPILLARYS2 FLEX PIERCING (Fully Automated Capillary Electrophoresis Instrument, Sebia Instruments, France). The electropherograms can be obtained (Figure 3). The concentration of cryoglobulins in the healthy is usually < 80 mg/L and cryoglobulins are polyclonal in the reference lab (reference interval: negative: cryoprecipitate specific volume < 0.4%, cryoglobulin protein concentration < 80 mg/L). For the patient, after receiving plasma exchange, blood purification, and methylprednisolone pulse therapy, his condition improved slightly. Due to the patient's reluctance to accept more treatment, further investigations for liver biopsy could not be performed.

## Diagnostic assessment

This patient had a history of alcoholic cirrhosis and was found to have renal impairment after admission. He presented with the classic Meltzer triad but had no symptoms of sensorimotor polyneuropathy and gradually developed the failure of the heart, the kidney, and the liver after the TIPS operation. Investigations revealed elevated serum BNP, serum creatinine, serum IgA, TT, APTT, and PT and reduced serum complement C4, C3, EGFR, hemoglobin, and platelets. In addition, 3.83 grams of protein, predominantly albumin, was seen in 24-h urine collection. The result of rheumatoid factor (RF) was negative. A previous study proposed that both the qualitative and quantitative associations of type II RF cryoglobulins with cryoglobulinemic vasculitis appear to occur mainly in patients infected with HCV than in non-infected individuals. The patient was not infected with HCV and HBV, so the result of RF may not be positive for him.

The results of the renal biopsy showed membranoproliferative glomerulonephritis and pathological changes of cryoglobulinemic glomerulonephritis. Immunofluorescence staining was positive for IgA1, IgG1, IgM, and C1. Bone marrow examination revealed polyclonal plasma cells, and mixed cryoglobulins (IgG κ and IgA κ) were identified in peripheral blood. A previous study suggested mixed cryoglobulinemic syndrome is diagnosed when a patient has typical organ involvement (mainly skin, kidney, or peripheral nerve) and circulating cryoglobulins (10). So, the patient was confirmed to be diagnosed as mixed cryoglobulinemia (type II–III) caused by alcoholic cirrhosis based on the medical history and the results of examinations (Table 1).

**Table 1:** Diagnoses assessment.

**Clinical signs**	**Laboratory findings**	**Histopathological findings**
Arthralgia	Mixed cryoglobulins	Polyclonal plasma cells disease
Skin purpura	Low C4 and C3	Active proliferation of hematopoietic cells
Asthenia	Proteinuria	Cryoglobulinemic glomerulonephritis
Liver involvement	BNP	
Renal involvement		
Heart failure		

## Discussion

Mixed cryoglobulinemia refers to the serum presence of a variety of cryoglobulins, which are defined as immunoglobulins that precipitate at temperatures of < 37°C. At present, only one study has reported that cryoglobulinemia was found in four out of 10 patients (one type II and three type III) with alcoholic liver cirrhosis (11), and literature on the case of type II–III mixed cryoglobulinemia caused by alcoholic liver cirrhosis has not been reported. Therefore, this is a rare case of type II–III mixed cryoglobulinemia with polyclonal plasma cell disease caused by rare etiology (alcoholic cirrhosis).

The main clinical manifestations of mixed cryoglobulinemia include palpable purpura (it suggests the presence of some form of vasculitis), renal disease, arthralgia or arthritis, non-specific systemic symptoms (including weakness), peripheral neuropathy, and hypocomplementary syndrome (decreased C4 levels are usually most prominent) (12, 13). Studies suggested that treatment should be modulated according to the underlying etiopathogenesis and the severity of clinical presentation rather than merely symptomatic relief. To date, no studies have reported any effective treatment. Patients with renal involvement should be treated according to two broad principles (immunosuppressive and etiology therapy) (10). This immunosuppressive therapy usually involves a short course of glucocorticoids (to suppress inflammation) and/or treatment with rituximab (to deplete B cells) (14, 15).

In this case, multiple organ failures (heart, kidney, and liver) occurred after TIPS surgery for alcoholic cirrhosis. Previous studies suggested that the involvement of kidneys occurs in 20% of mixed cryoglobulinemia patients (7). Based on the above evidence, we consider that the cause of multiple organ failure may be surgery and primary disease (mixed cryoglobulinemia). Therefore, it is important to identify the etiology of renal impairment timely, which is closely related to the prognosis of patients with liver cirrhosis. If the etiology of renal dysfunction for the patient can be identified as mixed polyclonal cryoglobulinemia timely, we will clarify the direction of treatment and treat this cause first using conventional immunosuppression and biological therapies, ultimately improving the success rate of treatment and reducing the fatality rate. Therefore, we should reflect on whether to treat the complication or the primary disease first when the patient diagnosed with mixed cryoglobulinemia has both liver cirrhosis and renal parenchymal damage.

## Conclusion

For a patient who has both liver cirrhosis and renal parenchymal damage, clinicians should increase awareness and facilitate early identification of the etiology of the renal disease, such as simple nephropathy, systemic disease, hepatorenal syndrome, or the rarely mixed cryoglobulinemia, as in the case of this patient, which is closely related to the method of treatment, the timing of treatment, and the prognosis of patients. As the clinical cases of mixed cryoglobulinemia are rare and its diagnoses are difficult, clinicians should keep in mind and evaluate carefully mixed cryoglobulinemia of liver cirrhosis and renal parenchymal. Early treatment of mixed cryoglobulinemia is important to alleviating multiple organ dysfunction, ultimately improving the success rate of therapy and reducing the fatality rate in a potentially life-saving therapy.

## Data availability statement

The original contributions presented in the study are included in the article/supplementary material, further inquiries can be directed to the corresponding authors.

## Ethics statement

Written informed consent was obtained from the individual(s) for the publication of any potentially identifiable images or data included in this article.

## Author contributions

JL, CL, and Q-JL: patient identification and article writing. TH, XQ, and YL: cytological and histopathological examination and image acquisition. J-LH and Q-MH: final drafting. All authors contributed to the article and approved the submitted version.
